# Partially coherent broadband 3D optical transfer functions with arbitrary temporal and angular power spectra

**DOI:** 10.1063/5.0123206

**Published:** 2023-04-03

**Authors:** Patrick Ledwig, Francisco E. Robles

**Affiliations:** Wallace H. Coulter Department of Biomedical Engineering, Georgia Institute of Technology and Emory University, Atlanta, Georgia 30332, USA

## Abstract

Optical diffraction tomography is a powerful technique to produce 3D volumetric images of biological samples using contrast produced by variations in the index of refraction in an unlabeled specimen. While this is typically performed with coherent illumination from a variety of angles, interest has grown in partially coherent methods due to the simplicity of the illumination and the computation-free axial sectioning provided by the coherence window of the source. However, such methods rely on the symmetry or discretization of a source to facilitate quantitative analysis and are unable to efficiently handle arbitrary illumination that may vary asymmetrically in angle and continuously in the spectrum, such as diffusely scattered or thermal sources. A general broadband theory may expand the scope of illumination methods available for quantitative analysis, as partially coherent sources are commonly available and may benefit from the effects of spatial and temporal incoherence. In this work, we investigate partially coherent tomographic phase microscopy from arbitrary sources regardless of angular distribution and spectrum by unifying the effects of spatial and temporal coherence into a single formulation. This approach further yields a method for efficient computation of the overall systems’ optical transfer function, which scales with *O*(*n*^3^), down from *O*(*mn*^4^) for existing convolutional methods, where *n*^3^ is the number of spatial voxels in 3D space and *m* is the number of discrete wavelengths in the illumination spectrum. This work has important implications for enabling partially coherent 3D quantitative phase microscopy and refractive index tomography in virtually any transmission or epi-illumination microscope.

## I. INTRODUCTION

Optical diffraction tomography is the premier label-free method to produce maps of refractive index within optically thick volumetric samples, typically in transmission^1^ but more recently also in epi-mode.^2^ A spatially coherent source is typically employed, and computational tomographic techniques can reconstruct a 3D map of phase and absorption (or a complex index of refraction) from a diverse variety of incident angles while projecting through a large number of focal planes at once. Partially coherent methods take an alternate approach by illuminating with a large number of incoherent incident angles at once, but only capturing the transmissive effects of one focal plane at a time, which is isolated due to the reduced coherence volume produced by the broad angular distribution of the light source. This allows the production of 3D maps of the index of refraction with quantitative accuracy using simple z-stage actuation as the only moving element, and reconstruction to be performed with a computationally inexpensive direct deconvolution.^3^

By deconvolving an imaging system’s optical transfer function (OTF) from a captured image, it is possible, in principle, to recover the phase and absorption of unlabeled specimens given only a knowledge of the imaging system’s acceptance pupil and source spatial and temporal frequency distribution.^4^ While producing an OTF for an axially-symmetric, monochromatic source can be performed rapidly by reducing dimensions,^5,6^ doing so with numerical accuracy for asymmetrical cases can be computationally expensive and infeasible for broadband sources. This limitation restricts the practical applicability of quantitative analysis to a limited set of well-controlled illumination parameters, such as annular phase-contrast^7,8^ or discrete collection of LEDs.^9^ Therefore, available technologies and theoretical frameworks fail to take advantage of widely available, low-cost partially coherent light sources that emit light over a broad range of wavelengths (e.g., lamps, LEDs, and thermal emitters) and that hold distinct advantages for microscopy over single-wavelength sources, such as improved spatial frequency support^10^ and temporal coherence gating.^11,12^ Sources that vary continuously or asymmetrically with angle and/or wavelength, such as scattered light through a diffusing medium,^2^ demand a more general treatment of the problem.

While partially coherent tomography typically achieves depth sectioning by making use of spatial coherence gating enabled by a broad angular spectrum, combining the effects of spatial and temporal coherence gating has been the motivation behind a number of recent tomographic technologies, including full-field optical coherence tomography,^12^ white light optical diffraction tomography,^13^ and reflection phase microscopy.^10^ The theoretical development presented in these works is tailored to their specific applications, relying on symmetries (such as axial symmetry) or sparsity (such as a discrete LED array) in illumination pattern and spectrum to produce a succinct expression for the relationship between the images formed and the object under investigation. However, many label-free techniques such as differential interference contrast,^14^ differential phase contrast,^15^ and quantitative oblique back illumination^2,16^ rely on continuous asymmetric illumination patterns to provide phase contrast.

In this work, we demonstrate a compact mutual coherence propagation kernel and employ it toward producing an efficient computational method to produce an OTF for sources with arbitrary spectral and angular power spectra. By analytically finding the set of broadband source wave vectors that can contribute to a given spatial frequency in a 3D intensity microscope image beforehand, we can compress the computation of optical transfer functions for practically any available source, streamlining computational image processing, and expanding the breadth of quantitative experimental techniques available to optical investigators.

## II. PARTIALLY-COHERENT BROADBAND 3D OPTICAL TRANSFER FUNCTION

To form a theoretical justification for this method, we start with an examination of the local state of coherence of a broadband field due to the incoherent contributions of emitting objects at a sufficient distance. As the second-order correlations of a stationary field themselves evolve according to coupled wave equations analogously to the field itself,^17^ spatial and temporal coherence are, therefore, fundamentally linked by laws of propagation^18^ (c.f. Sec. I of the supplementary material). It is through their mutual contributions that we are able to arrive at a concise formula for spatiotemporal coherence.

By constructing the mutual coherence within a spherical geometry, we can show that a surrounding volumetric source of arbitrary physical dimensions extends radially away from the near-field, and the object-space representation conveniently transforms into the 3D k-space representation of the field. This assumes a wide-sense stationary spatially incoherent volumetric source that is at a long distance from the observation region relative to both the maximum wavelength and the dimensions of the region of observation. In widefield microscopy, these conditions are met when illuminating with a source of any state of temporal coherence in the conjugate plane of the sample, the standard configuration for modern widefield microscopy,^19^ or, additionally, with a temporally incoherent source scattered through a diffusing volume that may be near the sample.^16^ More general sources, such as temporally coherent light scattered through a static diffuser, may require further treatment, such as that provided in Sec. II of the supplementary material for certain Schell-model sources.^20^

As shown in Sec. I of the supplementary material, a compact formulation of the source-integrated radiant spectral density of light impinging on the target object in a microscope can be found by propagating to the far-field in a spherical geometry, projecting the source distribution onto the unit sphere, and mapping temporal frequency onto the radial dimension in the 3D spherical reciprocal space $\vec{u}$, giving rise to the following 3D broadband analog to the van Cittert–Zernike theorem,

$$W\left(\vec{x},\nu \right)=\int S\left(\vec{u}\right)e^{i2\pi \vec{x}\cdot \vec{u}}\delta \left(|\vec{u}|-\nu \right)d^{3}\vec{u}.$$ (1)

Here, **x** represents 3D coordinates at the object, and **u** represents a 3D Fourier plane-wave space where the radial direction $\hat{u}$ maps to the direction of propagation. Notably, the radiant cross-spectral density, W (Fourier pair with the mutual coherence, Γ), depends only on three spatial dimensions (instead of six) due to a shift invariance in the local reference frame. In addition, due to the stationary statistics of the problem, we scale time by the speed of light and, therefore, represent frequency in units of inverse distance as linear wavenumber $\nu =\frac{k}{2\pi }$ (which we will refer to as wavenumber), to which we map the radius in reciprocal space |**u**|. The term $S\left(\vec{u}\right)$ represents the spatiotemporal spectral intensity of the source, describing its radiant power as a function of direction and wavenumber in the 3D Fourier space **u**, and is defined in detail in the supplementary material in Sec. I. We arrive at the result that the angular spectrum of the source spectral intensity $S\left(\vec{u}\right)$ contains sufficient information to give rise to the cross-spectral density of the illuminating light at the target, with a delta function isolating the interaction of individual temporal frequencies (*ν*). Equation (1) expresses the propagation of mutual coherence for a broadband source in a succinct expression, is evaluated with a single volumetric integral that resembles a frequency-sifted 3D Fourier transform, and forms the conceptual basis for the treatment presented in the rest of this work. Here and in the supplementary material, we define and apply this relationship in the context of a general imaging system, allowing us to produce a general-purpose 3D OTF for systems with arbitrary spatiotemporal source spectra and to compute them efficiently.

**FIG. 1.**
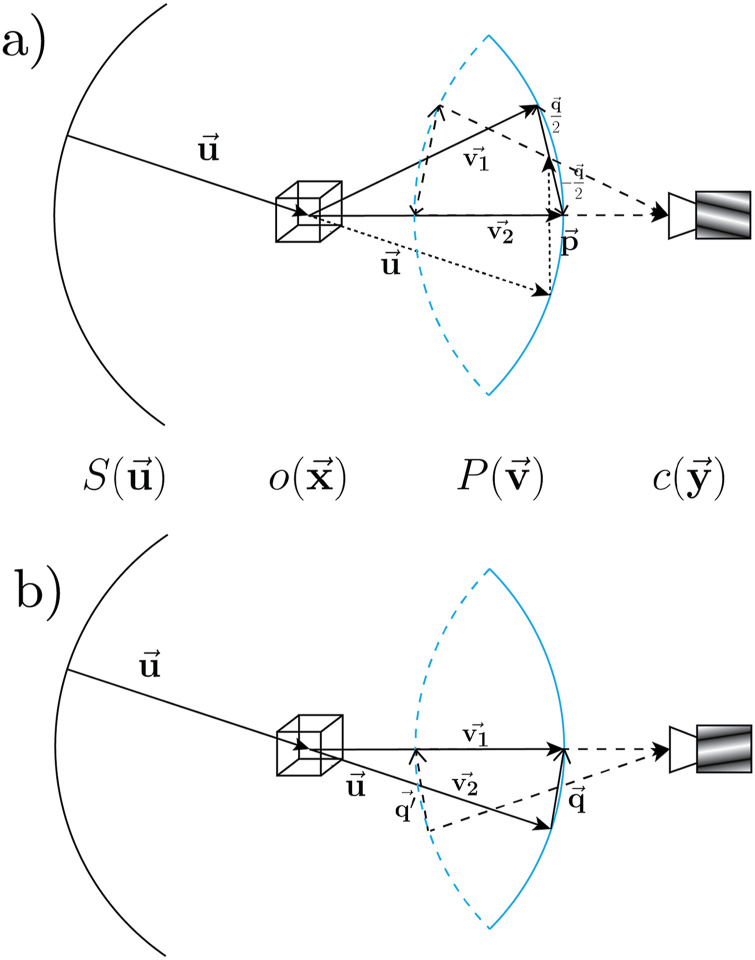
Representative diagrams depicting general transmission microscopy in a spherical far-field geometry. The source $\left(S\left(\vec{u}\right)\right)$ and pupil $\left(P\left(\vec{v}\right)\right)$ are represented in Fourier space as concentric circular segments (black and blue, respectively) equidistant from the object $\left(o\left(\vec{x}\right)\right)$ representing surfaces of equal wavelength. An intersecting dashed circular segment colored in blue represents the far-field of the camera, with fields collected by the blue segment, inverted onto the dashed segment, and relayed to the camera. Arrows with filled triangular tips represent field wave vectors with length proportional to wavenumber *ν*. Arrows with wedged tips represent standing waves, which can contribute to an image. The dashed lines represent fields relayed to the camera, and the dotted lines represent non-physical variables. (a) Diagram of representative geometry of the 3D Hopkins equation as described in Eq. (2). (b) Diagram of the linearized scenario described by the second term of Eq. (3).

By taking this approach, we propagate a cross-spectral density with wavelength selection imposed by the delta function in Eq. (1). Similarly, we can propagate arbitrary fields from the object out to the pupil plane and then again to the camera, while maintaining a concise form for the equation for the intensity image formed at the camera of a microscopic imaging system. Further details are offered in Sec. III of the supplementary material. By making use of the same line of reasoning due to propagation in a spherical geometry as was used with the treatment of the mutual coherence [see Fig. 1(a)], we produce a 3D broadband analog of the bilinear Hopkins’ equation for image intensity,

$$\begin{array}{ll} \tilde{I^{c}}\left(\vec{q}\right)= & \iint S\left(\vec{u}\right)O\left(\vec{p}+\frac{\vec{q}}{2}\right)O^{*}\left(\vec{p}-\frac{\vec{q}}{2}\right)P\left(\vec{u}+\vec{p}+\frac{\vec{q}}{2}\right)\\ & \times P^{*}\left(\vec{u}+\vec{p}-\frac{\vec{q}}{2}\right)\delta \left(\left|\vec{u}|-|\vec{u}+\vec{p}+\frac{\vec{q}}{2}\right|\right)\\ & \times \delta \left(\left|\vec{u}|-|\vec{u}+\vec{p}-\frac{\vec{q}}{2}\right|\right)d^{3}\vec{u}d^{3}\vec{p}. \end{array}$$ (2)

Here, intensity at the camera $\tilde{I^{c}}$ is evaluated in 3D Fourier space $\vec{q}$ as a convolution of the object transmission spectrum *O* with a filtering quantity formed by the convolution of the source (*S*), 3D pupil functions (*P*), and sampling delta functions over the source spatial frequency $\left(\vec{u}\right)$. As shown in the supplementary material, Sec. III, the explicit dependence on *ν* vanishes as it is naturally incorporated into the outward propagating fields $\vec{p}$. In addition, while biological samples are generally weakly absorbing and minimally dispersive in the visible and near infrared regions of the spectrum,^21^ the theory can be adapted to accommodate variation due to dispersion or variable absorption in situations where it may apply. Here we only consider the former case and leave the latter for future work.

This is only an intermediate result, but it is important to note the difference between Eq. (2) and the corresponding quasi-monochromatic counterpart well-established in the literature.^22^ The latter was developed with the implication that the results may be extended to a scenario with a broadband source by re-computing at each desired wavelength and integrating over the desired source spectrum.^23^ While this approach is clearly valid due to superposition, it may be computationally difficult or infeasible for certain non-reducible experimental conditions. By incorporating a more generalized broadband source from the inception, we find that the resulting intensity integral is more concise in its formalism and comprehensible in its description, allowing for the important insights which gave rise to the compressed computational approach described later in this work.

While Eq. (2) is of interest to phase-space optics research,^24^ it is computationally dense, and for the purposes of this work, it is an intermediate result that leads to the production of a 3D OTF, given by Eq. (3) (see the supplementary material, Sec. III, for the derivation). This result mirrors the formulation in the landmark work by Streibl,^3^ but once again with the implicit inclusion of an arbitrary source bandwidth,

$$\begin{array}{ll} {\tilde{T}}_{\alpha /\phi }\left(\vec{q}\right)= & \int S\left(\vec{u}\right)\left[{\left|P\left(\vec{u}-\vec{q}\right)\right|}^{2}\delta \left(\left|\vec{u}|-|\vec{u}-\vec{q}\right|\right)\right.\\ & \pm \left.{\left|P\left(\vec{u}+\vec{q}\right)\right|}^{2}\delta^{*}\left(\left|\vec{u}+\vec{q}|-|\vec{u}\right|\right)\right]d^{3}\vec{u}. \end{array}$$ (3)

Here, the subscripts *α* and *ϕ* refer to the absorption and phase signals, respectively. The arguments of the delta functions each describe a plane in $\vec{u}$ with an orthogonal vector $\pm \vec{q}/2$. This non-paraxial 3D phase and amplitude transfer function is similar to those reported in the literature,^2,8^ with the key distinctions being the implicit extension to a broad spectrum and consequently the form of the argument of the sifting delta functions. In addition, the assumption here is that the object is slowly varying rather than weakly reflecting. The former, in practice, provides a transfer function that is nearly equivalent to that produced by the weak object assumption but additionally allows the consideration of dark-field illumination terms, where source numerical apertures (NAs) exceed the pupil NA, and is based on the Rytov approximation, which is considered to be more valid in biological samples.^5,25^

The Hermitian symmetry of intensity imaging demands two wave vectors with equal and opposite projections on the camera [see Fig. 1(a)], and the sampling surfaces defined by the delta functions in Eq. (3) represent the collection of pairs of source field vectors equidistant from each other in the direction of the scattering object vector $\vec{q}$. By analogy, the sampling surface of the spectral density itself in Eq. (1), when interacting with the object, corresponds to the individual field vectors equidistant from the scattering vector (i.e. of the same wavenumber), giving rise to the Ewald sphere. Due to the coordinate change in the argument of the delta function from Eq. (1), the valid sampling surface in Eq. (3) changes in form from a sphere to a plane, so we refer to it as the Ewald plane. This plane defines the set of valid field interactions, each of which contributes incoherently to the total intensity of a given spatial frequency vector $\vec{q}$ in the 3D image space, regardless of wavelength. The Ewald planes select these fields for a given spatial frequency on the camera, and the resulting component in the linear 3D transfer function corresponds to the sum total of all incident light source fields that contribute to said spatial frequency. The amplitude signal is given by the sum of the two sampling Ewald planes selected with the integration over the delta functions in Eq. (3), while the phase signal is given by the difference.

## III. COMPRESSION OF COMPUTATION

In order to evaluate Eq. (3) quickly and efficiently, it is necessary to first determine the boundaries of the intersections of the Ewald planes with the source and pupil distributions so that a parsimonious sum may be taken over only the pre-determined non-zero regions. The angular dependence on acceptance of the source and pupil imposes a cone surface delineating their boundaries, whose apical angle is determined by their respective entrance NAs. The source and pupil cones may be centered at the origin. In addition, minimum and maximum temporal frequencies can be defined for a truncated effective spectrum, whose boundaries are given as spheres centered at the origin. The described geometry is further explored in the supplementary material, Sec. IV A.

**FIG. 2.**
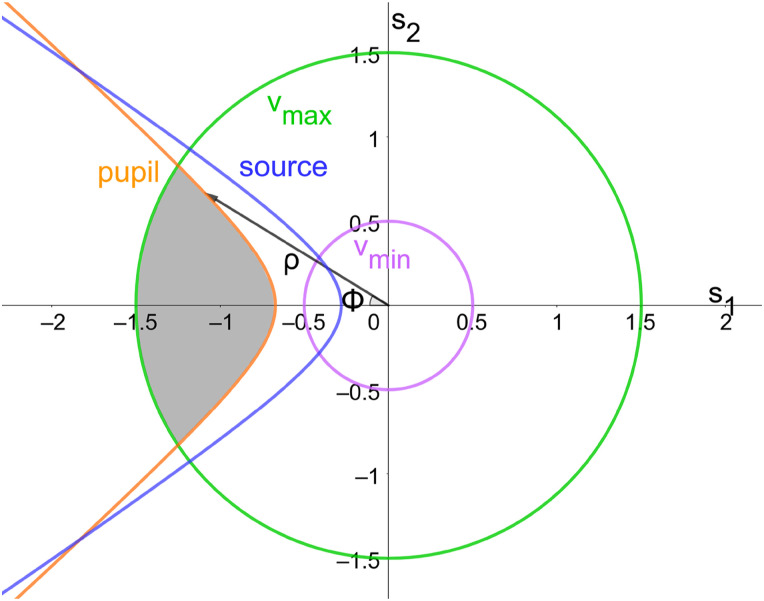
Sampling plane with traces of the exterior extent of the four limiting volumes, source (blue), pupil (orange), maximum frequency (green), and minimum frequency (magenta). The contours are plotted on axes *s*_1_ and *s*_2_, which represent the Cartesian coordinates in the frame of reference of an individual sampling plane, and the corresponding polar coordinates as *ρ* and Φ. The shaded region indicates the area of integration.

The intersections of the Ewald planes with the source and pupil boundaries form hyperbolas (or ellipses in some cases), and their intersections with the frequency limits form circles. It is, therefore, possible, using the methods detailed in the supplementary material, Sec. IV B, to analytically determine the boundaries of integration in the frame of reference of the intersecting plane. In polar coordinates on the surface, the radial extent of the integration will be determined by the frequency limits, and the angular extent will be determined by the pupil hyperbola on the left-hand side of the plane. Figure 2 demonstrates an example of the boundaries projected onto an intersecting plane, with the shaded region indicating the region of integration.

The value of the function to be integrated is determined by the product of the source spatial spectrum, temporal spectrum, and the appropriate Jacobian of transformation (see the supplementary material, Sec. V). The computation can be most conveniently performed in polar coordinates on the 2D sampling plane. The radial limits of integration are determined by the temporal spectrum, given trivially as

$$\rho_{\min}^{\max}=R\left\{\sqrt{{\left(\nu_{\min}^{\max}\right)}^{2}-q^{2}/2}\right\}.$$ (4)

The hyperbolic limits can be found with algebraic geometry and then the interior region by acquiring the logical intersection of the two hyperbolic regions. This analysis leads to a numerical formula for the surfaces of intersection defined by the angle Φ as shown in Fig. 2 (for details, see the supplementary material, Sec. IV B),

$$\begin{array}{ll} \cos\Phi_{o/i}=\; & \frac{-q}{2q_{x}\rho }\left(\sqrt{\rho^{2}+\frac{q^{2}}{4}}\left({\left(1+\alpha_{P}^{2}\right)}^{-\frac{1}{2}}+{\left(1+\alpha_{S}^{2}\right)}^{-\frac{1}{2}}\right)\right.\\ & +\left.\left|\sqrt{\rho^{2}+\frac{q^{2}}{4}}\left({\left(1+\alpha_{P}^{2}\right)}^{-\frac{1}{2}}-{\left(1+\alpha_{S}^{2}\right)}^{-\frac{1}{2}}\right)\mp q_{3}\right|\right), \end{array}$$ (5)

where the subscripts *o* and *i* are used to distinguish the Ewald surface of integration, referring to the surface with the outward-facing and inward-facing normal vectors, which determine if the shift was in the positive or negative $\frac{\vec{q}}{2}$ direction. Furthermore, *α*_*S*_ and *α*_*P*_ represent the source and pupil NA, respectively, and for notational convenience $q=|\vec{q}|$ and $q_{x}=\sqrt{q_{1}^{2}+q_{2}^{2}}$.

With the limits of integration determined by Eq. (5), the formula for computing the OTF [Eq. (3)] can be condensed to

$$\begin{array}{ll} T{\left(\vec{q}\right)}_{\alpha /\phi }= & \int_{\Phi_{o}}^{2\pi -\Phi_{o}}\int_{\rho_{\min}}^{\rho_{\max}}\frac{|\vec{u}\left(s_{o}\right)|S\left(\vec{u}\left(s_{o}\right)\right)|P\left(\vec{u}\left(s_{i}\right)\right){|}^{2}}{q}d^{2}s_{o}\\ & \pm \int_{\Phi_{i}}^{2\pi -\Phi_{i}}\int_{\rho_{\min}}^{\rho_{\max}}\frac{|\vec{u}\left(s_{i}\right)|S\left(\vec{u}\left(s_{i}\right)\right)|P\left(\vec{u}\left(s_{o}\right)\right){|}^{2}}{q}d^{2}s_{i}, \end{array}$$ (6)

where Φ_*o*/*i*_ refers to the corresponding sectioning plane, with either the outward or inward normal, and is computed with the inverse cosine of Eq. (5). In addition, the general 2D vectors **s**_**o**_ and **s**_**i**_ are being used for brevity represent polar coordinates on the respective surfaces, according to *d*^2^**s** = *ρ dρ d*Φ.

In summary, for each non-zero point in the image spatial frequency spectrum, the source spatiotemporal Fourier space intensity distribution is sampled over a subset of locations that represent the wavelengths and angles of incidence that may contribute incoherently to the given spatial frequency on the camera. As shown by the theoretical treatment presented here and in the supplementary material, this subset falls on a plane intersecting the source and pupil Fourier space distributions, which are themselves described by cones due to the angular selection of a finite acceptance aperture. The extent of possible contributing plane waves can, therefore, be determined beforehand with the analytic geometry of conic sections, treated more thoroughly in the supplementary material. Once determined, the Fourier space of the source may be sampled over the valid region at a rate determined by the user, which depends on the smoothness of the source distribution. This decouples computation cost from 3D image resolution for all but the bare minimum needed to produce a 3D OTF, and re-couples it with the sampling rate over a smoothly varying function, which in practice greatly reduces computation time for physical sources and large datasets.

This is the main result of this letter and describes a numerical and analytical procedure to produce a non-paraxial 3D OTF for a transmission or epi-mode imaging system for an arbitrary source, regardless of bandwidth, symmetry, or the state of temporal or spatial coherence, efficiently and accurately. We gain this advantage by analytically establishing the limits of integration that permit us to consider only non-zero values in the source distribution and by centering the evaluation for a single output coordinate of $\vec{q}$, again, considering only non-zero outputs. Furthermore, there is no reliance on 3D Fourier transforms or convolutions, as the calculation is pared down to its bare essentials. We, therefore, improve upon the numerical procedure that was previously conceived as a direct convolutional implementation of a version Eq. (3) for monochromatic sources only.^26^

## IV. RESULTS AND IMPLICATIONS

Previously, producing a 3D partially coherent OTF for arbitrary angular distributions would involve discretizing a source angular spectrum and centering computation on a single incident source direction and convolving spherical caps representing the pupil and source distributions in 3D Fourier space.^26^ However, even for a single wavelength, this approach comes at a higher computational cost, demands increased source resolution for increased accuracy, produces aliasing artifacts in regions of high spatial frequency, and, if necessary, demands repeat computation over each desired discrete wavelength over the source temporal spectrum.^23^

The computational complexity of this approach scales according to $O\left(N_{\nu }N_{x}^{2}N_{y}^{2}\right)$, where *N*_*ν*_ is the spectral resolution and *N*_*x*_ and *N*_*y*_ represent data size in the lateral dimensions. This scaling is due to contributions from both the temporal spectrum computation and the 2D source and pupil resolutions, which are convolved as spherical shells in 3D space. Here, the z resolution affects the accuracy of the produced 3D OTF, with artifacts arising at high spatial frequencies due to the discrete nature of the convolution. This can be compensated by over-sampling the source or pupil distributions. The scaling is further compounded by the fact that, in microscopy, it is typical for the lateral dimensions to have a much higher resolution than the axial dimension, making this dependence particularly challenging for high-resolution imaging.

With the approach presented in this work, this same task scales according to *O*(*N*_*x*_*N*_*y*_*N*_*z*_), due only to the resolution of the image in the 3D spatial frequency coordinates at the camera. The computational cost of the surface integral has been coupled with the precision of the numerical integral, which is tied to the smoothness of the source distribution in 3D k-space. Many practical sources are smooth in k-space, meaning that the Ewald plane can be sampled relatively sparsely while still providing consistent and accurate results for a broad spectrum in *ν*. More details about the implementation can be seen in the supplementary material (Fig. S9), and example code will be made available on the lab website.

**FIG. 3.**
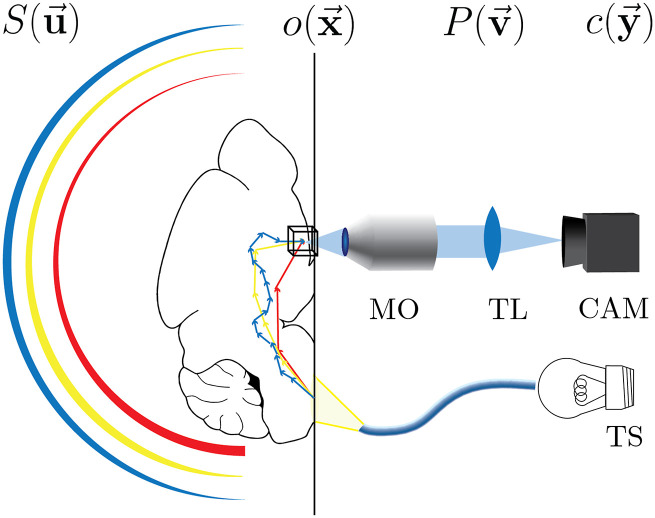
Representative diagram of source-detector geometry simulated in part IV, aligned in agreement with the diagrams of Fig. 1. Incoherent light from a thermal source (TS) is passed obliquely through a highly scattering tissue specimen, with lower wavelengths scattering more diffusely due to the medium’s wavelength-dependent scattering properties. The light impinging on the target varies continuously in color and power across the angular spectrum like a dawn-lit sky, making conventional OTF production computationally cumbersome. The line-widths of the constant wavelength circular segments of $S\left(\vec{u}\right)$ qualitatively represent the resulting spectral variation in photon directional tendency in the ensemble and, therefore, angular intensity at the target. The microscope objective (MO) forms the acceptance pupil, and the tube lens (TLs) relays the image onto the camera (CAM). The distances depicted are not proportional.

Efficiently producing arbitrary broadband 3D transfer functions streamlines processing for commonly-used illumination configurations and enables quantitative analysis of unconventional experimental scenarios, expanding the breadth of available tools for transmission and epi-mode microscopy and tomography. As an example, in Fig. 3, we demonstrate a scenario in which a thermal source is incident on biological tissues for epi-illumination , which requires both an arbitrary asymmetric frequency-dependent source angular distribution and an arbitrary incident power spectrum. In the visible regime, this represents a source whose intensity and color vary with incident angle, a scenario to which this work provides unparalleled quantitative computational access. This allows quantitative phase and absorption contrast calculations in arbitrary systems and enables optimization of non-ideal systems. This concept, for instance, is relevant to imaging modalities using diffusely-scattered light as an illumination source, such as quantitative oblique illumination microscopy,^16,27–29^ which may be configured with a broadband illumination source in order to provide an additional resource for shaping the effective system transfer function.

A readily available broadband light source may be thermal, for example, and, therefore, span a broad range of temporal frequencies and may approach the objective focus at preferentially oblique angles, producing asymmetry in the effective source distribution. Further complicating the picture, the scattering medium itself, e.g., tissue, may have wavelength-dependent variation in scattering parameters, changing the obliquity and, therefore, the effective source spatial frequency distribution as a function of temporal frequency. The approach presented in this work handles this three-fold asymmetry with no more difficulty and even lower computational cost than using a previous method with a simple monochromatic illumination.

**FIG. 4.**
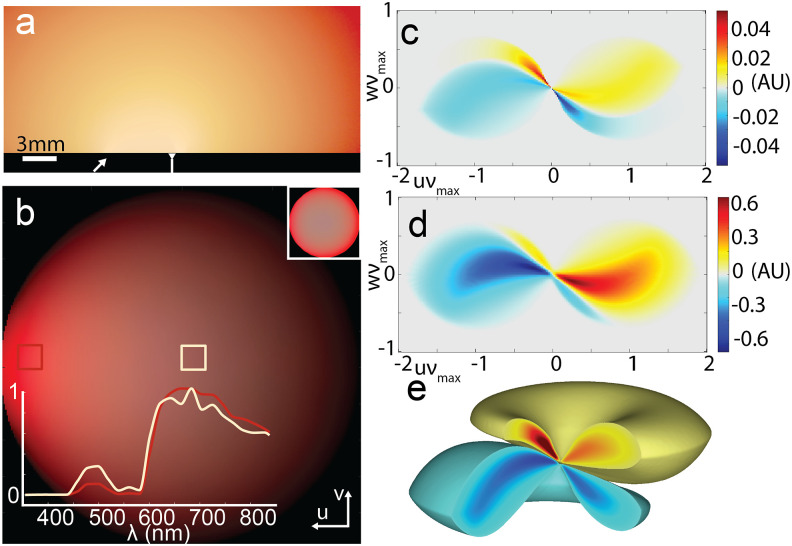
Diffusely scattered 6000 K white light transport through brain as a model of angle- and spectral-dependent illumination conditions. (a) Relative log-visitation of white light in brain. Color scale represents spectrum with human-perceived color. Scale bar is 3 mm, and source (arrow) is positioned 7 mm laterally from the objective (reversed-tip arrow). (b) Received spatiotemporal spectrum at focal plane of objective, projected upper hemisphere of collected angle, with human-perceived color representing relative spectrum. *Inset*, *bottom* *left*: Relative spectral intensity in nm collected from forward-travelling (red) and overhead (beige). *Inset*, *top right*: Intensity-normalized spatiotemporal spectrum demonstrating relative human-perceived color differences without darkening from intensity. (c) Broadband transfer function computed from spatiotemporal spectrum represented in (b) with pupil NA = 0.95. Scale of z-x axis is in units of wavelength-normalized spatial frequency, scaled to the lowest wavelength present (*ν* = *ν*_max_). (d) Single-wavelength transfer function using only the mean transmitted wavelength. (e) 3D representation of spectrum shown in (c).

Figure 4 shows the photon log-visitation distribution using a Monte Carlo light transport simulation of light scattered sub-diffusely due to small source-detector separation and an oblique incident illumination angle in simulated brain tissue.^16,21,30^ Spectral-dependent absorption and scattering in the tissue produces an angular preference for certain wavelengths, giving rise to an overhead hemispherical projection with striking variations in color, just as Rayleigh scattering gives rise to color variations in the sky during sunset, which can be mapped on to $S\left(\vec{u}\right)$ in Eq. (1). As shown in Figs. 4(c) and 4(e), this illumination scheme flattens out the variation in the main lobes of the transmitted transfer function relative to the single-wavelength case [Fig. 4(d)]. This can be advantageous for reconstructing an image with deconvolution, as it not only provides a more accurate and uniform representation of the system’s sensitivity to the object’s spatial frequencies, but it also avoids dampening an object’s high frequency content.^31^

With the method presented, computing a broadband transfer function [e.g., Figs. 4(c) and 4(e)] takes no more time than if it were used to produce a monochromatic transfer function [e.g., Fig. 4(d)] and takes less computational time than using the previous conventional convolutional methods, even with monochromatic illumination.^2,22,23,26,32^ Using the same resolution (256 × 256 × 128 pixels), we were able to produce the broadband transfer function in 20 seconds on an Intel Core i7 7800X 3.5 GHz CPU using GPU parallelization with a GTX 1080 Ti. By contrast, the monochromatic transfer function, computed with the same approach used in previous work and elsewhere in the literature,^2,26,32^ by accumulating transfer functions from scaled source point-wise across the entire source angular spectrum (as in the conventional convolutional approach), took 1 h and 20 min on the same hardware, also making use of GPU acceleration. Furthermore, to achieve a faithful representation of a transfer function produced from a broad spectrum, the single wavelength transfer function would have to be repeated for each wavelength across the spectrum, making it an approach that is not computationally feasible in any case. Other experimental use-cases and computational benchmarks are provided in the supplementary material, Sec. VI, including phase-contrast with uneven illumination through a diffuser and transfer function synthesis with black-body illuminations of varying temperatures. The former’s continuous variation in effective angular intensity and the latter’s wavelength dependence would both otherwise demand considerable computation, inhibiting prototyping and iteration of experiment and device design.

## V. CONCLUSION

In conclusion, we have presented a method to produce the 3D optical transfer function of a microscope with arbitrary angular and temporal spectra in a computationally efficient and accurate manner, enabling partially coherent 3D quantitative phase microscopy in virtually any transmission and epi-mode (non-reflection) microscope. By unifying the angular and temporal coherence into the 3D angular spectrum, this work advances deconvolution methods by allowing for quantitative analysis of microscopy in a wide range of illumination scenarios, enabling creative experimental methods that would otherwise be out of reach from quantitative analysis. Furthermore, this method allows for rapid turnaround of 3D OTFs, enabling, for example, the dynamic acquisition of time-variant and/or spatially-variant systems and non-linear computational techniques that may incorporate iterative updates to an estimated system OTF. Furthermore, the methods presented here may be extended into other computational problems in the field, such as those involving the phase space representation of the non-linear Hopkins equation, as shown in Eq. (2).

## SUPPLEMENTARY MATERIAL

The supplementary material contains more details involving the construction of the spatiotemporal mutual coherence, a generalization of the method for Schell-model source coherence, a detailed derivation of the broadband transmission microscope transfer function using the formalism defined and adopted in this work, a validation comparison to the quasi-monochromatic case, and a flowchart guide to efficient and precise numerical computation.

## Data Availability

The data that support the findings of this study are available from the corresponding author upon reasonable request.
